# Three-dimensional visualization improves the endoscopic diagnosis of superficial gastric neoplasia

**DOI:** 10.1186/s12876-021-01829-y

**Published:** 2021-05-28

**Authors:** Kazutoshi Higuchi, Mitsuru Kaise, Hiroto Noda, Kumiko Kirita, Eriko Koizumi, Takamitsu Umeda, Teppei Akimoto, Jun Omori, Naohiko Akimoto, Osamu Goto, Atsushi Tatsuguchi, Katsuhiko Iwakiri

**Affiliations:** 1 Department of Gastroenterology, Nippon Medical School, Graduate School of Medicine, 1-1-5, Sendagi, Bunkyo-ku, Tokyo 113-8603 Japan; 2 Department of Analytic Human Pathology, Nippon Medical School, Graduate School of Medicine, Tokyo, Japan

**Keywords:** Three-dimensional endoscopy, Two-dimensional endoscopy, Early gastric cancer, Cancer extent

## Abstract

**Background:**

Three-dimensional (3D) technology has been used in many fields, including flexible endoscopy. We evaluated the usefulness of 3D visualization for endoscopically diagnosing superficial gastric neoplasia.

**Methods:**

Twelve participants (4 novices, 4 trainees and 4 experts) evaluated two-dimensional (2D) and 3D endoscopic still images of 28 gastric neoplasias, obtained before ESD with white-light imaging (WLI) and narrow-band imaging (NBI). Assessments of the delineation accuracy of tumor extent and tumor morphology under 2D and 3D visualization were based on the histopathological diagnosis of ESD specimens. Participants answered visual analog scale (VAS) questionnaires (0–10, worst to best) concerning the (a) ease of recognition of lesion morphology, (b) lesion extent and (c) comprehensive endoscopic cognition under 2D and 3D visualization. The endpoints were the accuracy of tumor extent and morphology type and the degree of confidence in assessing (a)–(c).

**Results:**

The delineation accuracy of lesion extent [mean (95% confidence interval)] with WLI under 3D visualization [60.2% (56.1–64.3%)] was significantly higher than that under 2D visualization [52.3% (48.2–56.4%)] (*P* < 0.001). The accuracy with NBI under 3D visualization [70.3% (66.8–73.7%)] was also significantly higher than that under 2D visualization [64.2% (60.7–67.4%)] (*P* < 0.001). The accuracy of the morphology type with NBI under 3D visualization was significantly higher than that under 2D visualization (*P* = 0.004). The VAS for all aspects of endoscopic recognition under 3D visualization was significantly better than that under 2D visualization (*P* < 0.01).

**Conclusions:**

Three-dimensional visualization can enhance the diagnostic quality for superficial gastric tumors.

**Supplementary Information:**

The online version contains supplementary material available at 10.1186/s12876-021-01829-y.

## Background

Gastric cancer is the fifth-most commonly diagnosed cancer worldwide and the third-most common cause of cancer-related death [1]. Early detection of gastric cancer is key to reducing the rate of cancer death. Early gastric cancer (EGC) without lymph node metastasis can be cured by endoscopic submucosal dissection (ESD), and the quality of life after ESD is quite favorable [2, 3].

Detecting gastric cancer in an early stage and precisely delineating the cancer margin are necessary for a high cure rate by ESD. Novel technologies and devices have improved the quality of the endoscopic diagnosis. Optical image-enhanced technologies, such as narrow-band imaging (NBI), blue laser imaging and linked color imaging have facilitated the diagnosis of superficial gastric neoplasia [4–7]. The technology of three-dimensional (3D) visualization is currently being used in many fields, including movies, video games and show attractions in amusement parks, and has also been introduced to some medical fields.

The technique of 3D rigid endoscopy is clinically used in surgical fields and has enabled a more accurate and speedy procedure than a two-dimensional (2D) approach [8, 9]. Furthermore, this approach has been introduced in robotic surgery, enhancing the precision of minimally invasive surgery [10]. In addition, 3D flexible endoscopy has also been developed. In an ex vivo animal model of ESD, 3D visualization shortened the procedure time and decreased the adverse event rate compared to 2D, especially in trainees [11–13]. Recently, 3D flexible endoscopy was clinically applied in humans and reported to be feasible for ESD [14]. However, while several studies have recently addressed the efficiency of 3D flexible endoscopy in this procedure [15], very few clinical studies have evaluated the usefulness for making an endoscopic diagnosis.

The present study evaluated the usefulness of 3D visualization for determining the margin of superficial gastric neoplasia compared to 2D visualization in clinical practice.

## Materials and methods

### Enrolled lesions and endoscopic images

Twenty-nine superficial gastric neoplasias excised by ESD in Nippon Medical School Hospital from March 2018 to January 2019 were serially enrolled. Endoscopic still images of the enrolled lesions were obtained before ESD marking with 2D white-light imaging (WLI), 3D-WLI, 2D-NBI and 3D-NBI. The 2D and 3D images of each lesion were exactly same aside from being obtained via 2D or 3D visualization. This was possible because the 3D image set and corresponding 2D image set were both preserved when a still image was taken with the 3D endoscopy system. A set of representative 2D-WLI, 3D-WLI, 2D-NBI and 3D-NBI images from each lesion was extracted from these images and used in this study. After the endoscopic observation, all lesions were excised by ESD and pathologically evaluated, including an assessment of the tumor extent diagnosis. The pathological tumor margin was delineated on a color print of the representative 2D image of each lesion in advance.

### Examination protocol

**Fig. 1:**
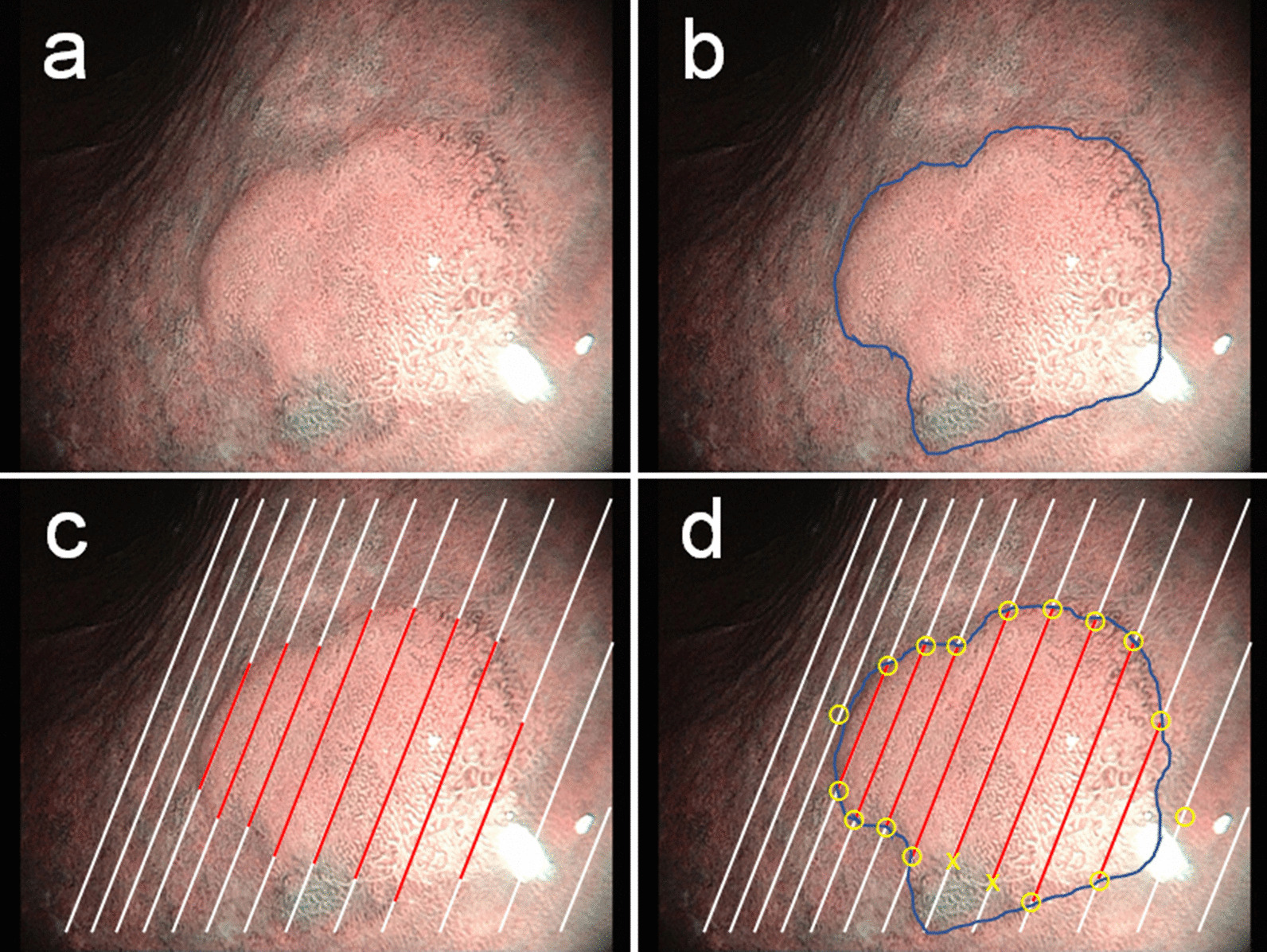
Definition of the concordance rate of lesion extent. **a** Endoscopic image of a gastric neoplasia using NBI. A superficial protruded-type lesion (adenoma) is evident on the lesser curve of the lower gastric body. **b** Delineation of the tumor margin is performed. The blue line is the tumor extent line drawn by a participant. **c** According to the histopathological findings, the tumor extent (red lines) is reconstructed on an endoscopic image. The white lines are the cutting lines of a resected specimen. **d** The calculation of the concordance rate of lesion extent. Yellow circles and yellow cross marks show coincident and non-coincident evaluating points, respectively. In this case, 16 of the 18 evaluation points were coincident, indicating a coincident rate of lesion extent of 88.9% (16/18)

**Fig. 2:**
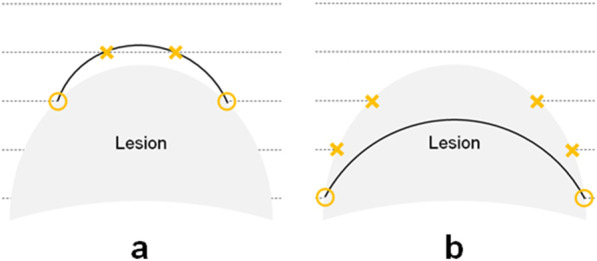
Definition of the evaluation points for outside the lesion. **a** If the drawn line passes through the cutting lines outside the actual tumor margin, the intersections are evaluated as incorrect points. The black line shows the drawn line and dotted lines show the cutting lines. The yellow circles and yellow cross marks show the correct and incorrect evaluation points, respectively. **b** If the drawn line passes inside the tumor margin, the evaluation points that the drawn line do not pass through on the lesion border are evaluated as incorrect points

The primary endpoint of this study was the accuracy of the tumor extent diagnosis assessed by the concordance rate between the pathological tumor margin and the endoscopically assumed tumor margin under 2D or 3D visualization. Study participants drew an endoscopically assumed tumor margin on a color print of a 2D image of an enrolled lesion while viewing the endoscopic still image on the video monitor under 2D or 3D visualization (Fig. 1a, b). The concordance between the pathological and drawn margins was then evaluated on preset lines of the color print that corresponded to the tissue section lines of 2-mm-wide strips of ESD specimens (Fig. 1c). If the pathological tumor margin and drawn margin matched on the preset line, then it was assessed as being correct. If they did not match, it was assessed as being incorrect (Fig. 1d). If the drawn line was outside the pathological margin and intersected with the next adjacent preset line, the number of these intersecting points was counted as evaluation points and, then these points were assessed as being incorrect (Fig. 2a). If the drawn line was inside the pathological margin and did not pass the preset line, the evaluation points that the drawn lines did not pass through were assessed as being incorrect (Fig. 2b). The concordance rate was calculated as the number of correct points divided by the number of total evaluation points on the lines. The concordance rate was used as an index of the accuracy of the lesion extent diagnosis.

Twelve study participants were blinded to the endoscopic findings and histopathological diagnoses of the enrolled superficial gastric neoplasias. These participants were 4 novices (young physicians with no endoscopic practice), 4 trainees (physicians with experience performing ESD in 30–300 cases) and 4 experts (endoscopists with experience performing ESD in over 300 cases and Board-certified Fellows of the Japan Gastroenterological Endoscopy Society). A crossover design for evaluating the 2D and 3D images was used to reduce bias.

**Fig. 3:**
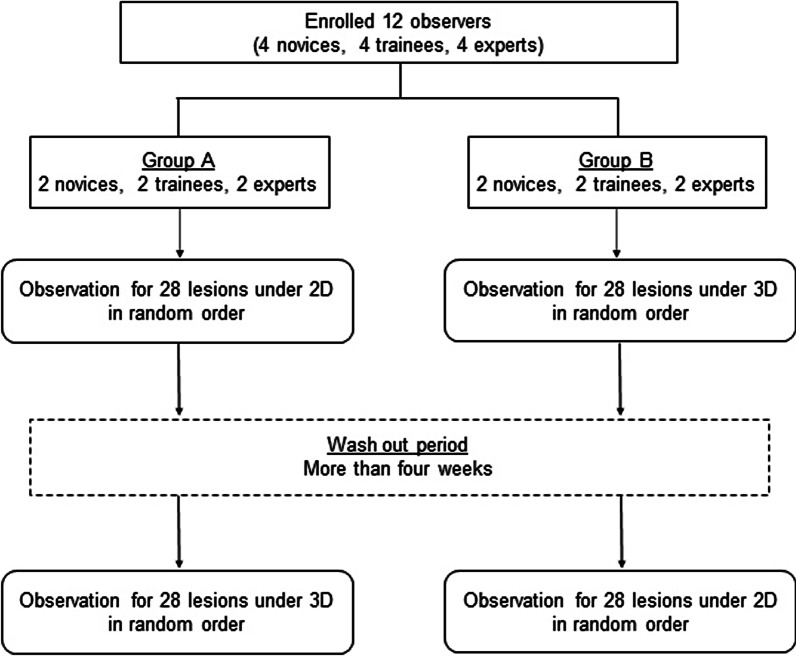
Flow chart showing the enrollment of the participants and evaluation of endoscopic images

The novices, trainees and experts were divided equally into two groups (A and B). Group A evaluated 2D images first, followed by 3D images, while group B evaluated 3D images first, followed by 2D images. To reduce the carry-over effect between 2 and 3D observation, the first and the second observations were interrupted by an interval exceeding four weeks as a wash-out period (Fig. 3). After finishing the delineation of the tumor margin, participants described the morphology type of the lesions and responded to visual analog scale (VAS) questionnaires concerning endoscopic lesion recognition, which was evaluated based on three aspects: (a) ease of recognition of lesion morphology, (b) ease of recognition of lesion extent and (c) ease of recognition of comprehensive endoscopic cognition. The VAS ranged from 0 to 10 (worst to best). If the endoscopists found the endoscopic observation very difficult, the rating was 0. If they found it very easy, the rating was 10. If they found it neither difficult nor easy, the rating was 5. The morphology type was evaluated according to the macroscopic type classification described in Japanese classification of gastric carcinoma [16]. In this study, we used the pathological morphology as the final morphology of tumors. The pathological typing of tumor morphology was done by pathologists, who macroscopically viewed the mucosal surface and also microscopically reviewed the lesion. Participants were given 30 s per lesion for the evaluation. During the time, they were able to observe the image of the lesion on the video monitor as many times as they wanted. After finishing the evaluation of one lesion, the next evaluation of another lesion was performed. The order of lesions to be displayed was randomly assigned. Participants evaluated WLI and NBI images of all enrolled lesions.

### Outcome measurement and sample size

The primary endpoint of this study was the coincidence rate of the lesion extent. The accuracy of morphology type and ease of endoscopic lesion recognition were secondary endpoints. The sample size was not calculated because this was the first clinical pilot study, and no similar studies were available for reference. Around 30 lesions had been tentatively assumed as the target enrollment number along with the numbers enrolled in previous 3D endoscopy studies that involved comparisons with 2D endoscopy.

### 3D endoscopy system

**Fig. 4:**
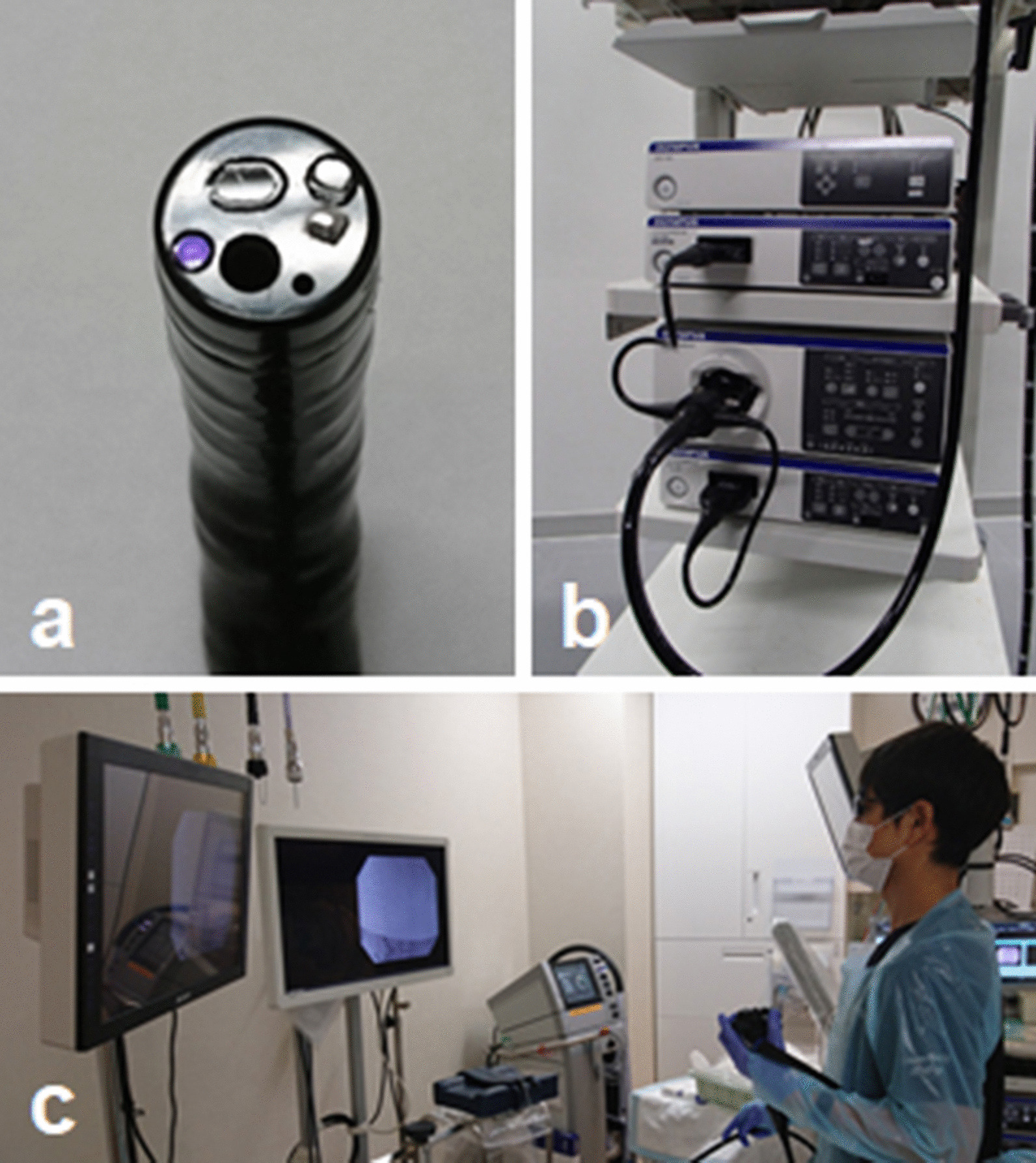
A 3D flexible endoscopy system. **a** Three-dimensional endoscope with two lenses and a forceps channel on its tip. **b** The 3D flexible endoscopy system consists of a 3D flexible endoscope, two video system centers, a 3D Visualization Unit, a light source and a 3D monitor. **c** The 3D image is visualized using the 3D monitor and 3D glasses

A 3D endoscope with stereoscopic optical system (GIF-Y0080; Olympus Medical Systems Corp., Tokyo, Japan) was used in this study (Fig. 4a). The electrical signal of the image obtained through each lens was transmitted to a video system center (EVIS EXERA III Video System Center, CV-190; Olympus Medical Systems Corp.) and synthesized in a 3D Visualization Unit (3DV-190; Olympus Medical Systems Corp.) (Fig. 4b). Endoscopists with 3D glasses were able to review the 3D images on a 3D monitor (LMD-2451MT; Sony, Tokyo, Japan) (Fig. 4c).

### Statistical analyses

All variables were presented as the mean and standard deviation. Between-group comparisons were performed using Wilcoxon’s signed-rank test for continuous variables and McNemar’s test for categorical variables. *P* values < 0.05 were considered to be statistically significant. All statistical analyses were performed using the SPSS statistical software package (version 25; IBM Corp., New York, NY, USA).

## Results

The en bloc resection rate by ESD of the 29 enrolled lesions was 100%. One enrolled lesion was excluded because no neoplasia was found on the resected specimen by a histopathological evaluation. Therefore, the endoscopic images of 28 lesions were ultimately evaluated by the participants, equating to112 images of 2D-WLI, 3D-WLI, 2D-NBI and 3D-NBI. A pathological examination revealed that the 28 excised lesions consisted of 26 EGCs (21 well- and moderately differentiated adenocarcinomas, 4 Signet-ring cell carcinomas and 1 poorly differentiated adenocarcinoma) and 2 gastric adenomas. The mean lesion size was 14.8 ± 7.6 mm. The morphology types were superficial protruded type in 4, flat type in 3 and superficial depressed type in 21 (Table 1).

**Table 1:** The characteristics of the lesions

En bloc resection rate, % (n)	100% (28/28)
Lesion size, mm	14.8 (7.6)
Specimen size, mm	44.0 (12.8)
*Location*	
Upper third, n (%)	3 (10.7%)
Middle third, n (%)	13 (46.4%)
Lower third, n (%)	12 (42.9%)
*Histological type*	
Well- and moderately differentiated adenocarcinoma, n (%)	21 (75.0%)
Signet-ring cell carcinoma, n (%)	4 (14.3%)
Poorly differentiated adenocarcinoma, n (%)	1 (3.6%)
Adenoma, n (%)	2 (7.1%)
*Morphology type*	
Superficial protruded type, n (%)	4 (14.3%)
Flat type, n (%)	3 (10.7%)
Superficial depressed type, n (%)	21 (75.0%)

Data are presented as the mean (standard deviation)

The concordance rate of the lesion extent with WLI under 3D visualization [60.2%, 95% confidence interval (CI) 56.1–64.3%] was significantly higher than that under 2D visualization (52.3%, 95% CI 48.2–56.4%) (*P* < 0.001), and that with NBI under 3D visualization (70.3%, 95% CI 66.8–73.7%) was also significantly higher than that under 2D visualization (64.2%, 95% CI 60.7–67.4%) (*P* < 0.001). There were no significant differences between group A and Group B on 2D-WLI, 2D-NBI, 3D-WLI and 3D-NBI, respectively (Additional file 1: Supplementary Table 1).

The subgroup analysis with the skill level of endoscopy is shown in Table 2. In the novices, the tumor extent diagnosis under 3D-WLI was significantly superior to that under 2D-WLI, but the difference was not significant under NBI. In the experts, the superiority of 3D over 2D visualization was significant under NBI but small under WLI. In the trainees, the superiority of 3D over 2D visualization was most significant with about 10% addition under WLI and NBI observation.

**Table 2:** The coincidence rate of lesion extent

	WLI			NBI		
	2D	3D	*P* value	2D	3D	*P* value
Overall	52.3% (48.2–56.4%)	60.2% (56.1–64.3%)	< 0.001	64.0% (60.7–67.4%)	70.3% (66.8–73.7%)	< 0.001
Novice	39.3% (31.9–46.7%)	50.8% (43.2–58.4%)	< 0.001	47.4% (41.5–53.4%)	49.1% (42.9–55.4%)	0.55
Trainee	53.8% (46.7–60.8%)	62.4% (55.6–69.2%)	0.001	68.0% (62.8–73.3%)	78.0% (73.1–82.9%)	< 0.001
Expert	63.9% (57.4–70.3%)	67.4% (60.8–74.0%)	0.055	76.8% (71.6–81.9%)	83.8% (79.4–88.2%)	< 0.001

Data are presented as the mean (95% confidence interval)

*WLI* white-light imaging, *NBI* narrow-band imaging, *2D* 2-dimensional, *3D* 3-dimensional

The subgroup analysis with the morphology type is shown in Table 3. In depressed type, the tumor extent diagnosis under 3D visualization with both WLI and NBI was more accurate than that under 2D visualization (WLI: 53.2% vs. 43.4%, NBI: 68.7% vs. 61.5%, respectively *P* < 0.001). In flat type, the superiority of 3D over 2D visualization was not found under WLI or NBI because the accuracy rates were poor under 3D visualization. In protruded type, the superiority of 3D over 2D visualization was found under NBI but not under WLI because the accuracy rates under 2D-WLI reached 95.8%.

**Table 3:** The comparison by the morphology of the accuracy in assessing lesion extent

	WLI			NBI		
	2D	3D	*P* value	2D	3D	*P* value
Protruded type	95.8% (93.2–98.4%)	97.2% (95.5–98.8%)	0.320	77.5% (70.5–84.5%)	83.2% (77.5–89.0%)	0.005
Flat type	47.9% (36.8–59.0%)	52.9% (40.4–65.3%)	0.127	57.3% (45.7–69.0%)	58.5% (46.2–70.8%)	0.879
Depressed type	43.4% (38.8–47.9%)	53.2% (48.5–57.9%)	< 0.001	61.5% (57.5–65.5%)	68.7% (64.6–72.8%)	< 0.001

Data are presented as the mean (95% confidence interval)

*WLI* white-light imaging, *NBI* narrow-band imaging, *2D* 2-dimensional, *3D* 3-dimensional

The accuracy of the morphology type diagnosis with NBI under 3D visualization (76.0%, 95% CI 71.0–80.0%) was significantly higher than that under 2D visualization (69.0%, 95% CI 63.0–74.0%) (*P* = 0.004). In contrast, there was no significant difference between 2D (66.0%, 95% CI 61.0–72.0%) and 3D (71.0%, 95% CI 66.0–76.0%) visualization under WLI (*P* = 0.104) (Table 4).

**Table 4:** The accuracy of the morphology type

	WLI			NBI NBI		
	2D	3D	*P* value	2D	3D	*P* value
Overall	66.0% (61.0–72.0%)	71.0% (66.0–76.0%)	0.104	69.0% (63.0–74.0%)	76.0% (71.0–80.0%)	0.004
Novice	49.0% (39.0–59.0%)	60.0% (50.0–69.0%)	0.043	52.0% (42.0–61.0%)	67.0% (58.0–76.0%)	0.005
Trainee	76.0% (68.0–84.0%)	79.0% (71.0–87.0%)	0.648	77.0% (69.0–85.0%)	83.0% (76.0–90.0%)	0.118
Expert	74.0% (65.0–83.0%)	74.0% (65.0–83.0%)	1.0	77.0% (69.0–85.0%)	77.0% (69.0–85.0%)	1.0

Data are presented as the mean (95% confidence interval)

*WLI* white-light imaging, *NBI* narrow-band imaging, *2D* 2-dimensional, *3D* 3-dimensional

In the VAS, the (a) ease of recognition of lesion morphology, (b) ease of recognition tumor extent and (c) ease of recognition of total endoscopic cognition were higher under 3D visualization than under 2D visualization with both WLI and NBI (*P* < 0.001, respectively) (Table 5). The comparison by skill level is shown in Additional file 1: Supplementary Table 2.

**Table 5:** The comparison of the ease of endoscopic lesion recognition

	WLI		*P* value	NBI		*P* value
	2D	3D		2D	3D	
Lesion morphology	4.78 (2.75)	7.03 (2.84)	< 0.001	5.85 (2.28)	7.68 (2.34)	< 0.001
LESION extent	4.18 (2.75)	6.05 (2.86)	< 0.001	5.07 (2.49)	6.76 (2.53)	< 0.001
Comprehensive endoscopic cognition	4.28 (2.81)	6.21 (2.98)	< 0.001	5.35 (2.42)	7.18 (2.54)	< 0.001

Data are presented as the mean (standard deviation)

*WLI* white-light imaging, *NBI* narrow-band imaging, *2D* 2-dimensional, *3D* 3-dimensional

## Discussion

The present study revealed that the endoscopic recognition of the lateral margin of superficial gastric neoplasia under 3D visualization was more accurate and easier than that under 2D visualization, particularly in superficial depressed type. In addition, morphology recognition in NBI was easier under 3D than 2D visualization. To our knowledge, this is the first study to show the usefulness of 3D visualization for the endoscopic diagnosis of gastric neoplasia in clinical practice.

ESD is an excellent minimally invasive treatment for EGC without lymph node metastasis, and the range of indications has expanded with the development of technologies and devices associated with this technique. However, an inaccurate recognition of the tumor margin may result in incomplete resection with a positive margin for tumor cells, which can result in local recurrence. The precise recognition of differences in superficial morphology under 3D visualization enables the more accurate delineation of the tumor margin and may thus enhance the curability of ESD for superficial gastrointestinal neoplasia.

Chromoendoscopy with indigo carmine has enabled the accurate diagnosis of the horizonal margin in 80% of EGCs [17, 18]. It was particularly highly effective for protruded type 0-IIa and 0-I and depressed type 0-IIc lesions, although successful delineation was achieved in only 32.1% of EGCs for flat type 0-IIb. Iizuka et al. reported that the addition of acetic acid spray before dispersing indigo carmine clarified the tumor margin compared with only indigo carmine [19]. Several studies found that magnifying endoscopy with NBI (M-NBI) was more useful than chromoendoscopy for delineating the lateral margin of EGC [20]. M-NBI successfully defined the lateral margin of 72.6% of EGCs with unsuccessful delineation on chromoendoscopy [17]. In contrast, a multicenter randomized study revealed that chromoendoscopy and M-NBI were clinically equivalent for determining the horizonal margin of EGC [21]. In the present study, the delineation of the tumor margin for superficial depressed type was more accurate under 3D than 2D visualization with both WLI and NBI. Three-dimensional visualization can identify subtle changes in the gastric mucosal epithelium by providing depth perception. In contrast, there was no significant difference between 2 and 3D visualization with WLI for superficial protruded type, although the accuracy of the lesion extent under 3D visualization was greater than that under 2D visualization with NBI, possibly due to the fact that the concordance rate of the lesion extent with WLI exceeded 95% even with 2D, meaning that recognizing the lateral margin in the protruded type is relatively easy.

There were only three flat-type EGCs, and while they were difficult to statistically evaluate, the 2D and 3D visualization results were equivalent. A previous study in which resected ESD specimens of superficial gastric tumor were evaluated also reported that 3D visualization enabled the accurate delineation of the lateral margin for type 0-IIc but not for type 0-IIb [22]. Due to the lack of any marked difference in the mucosal height from the surrounding mucosa in flat-type EGC, the accurate recognition of the tumor extent, even with various devices and techniques, is quite challenging. However, the accuracy of the lesion extent and the morphology type was higher with NBI than with WLI by 10% and 5% under 2D and 3D observation, respectively. Although the comparison of the still images of WLI and NBI was not impartial because both the WLI and NBI for each lesion were not same in this study, NBI may result in the better recognition of gastric lesions. Therefore, 3D-NBI observation is considered the most useful modality for detecting gastric cancerous lesions and recognizing the tumor extent at present.

The endoscopic diagnosis of EGC is usually affected by the experience and skill level of the endoscopist. Indeed, the accuracy of the tumor extent and morphology type in experts was quite high compared to that of the trainees under both 2D and 3D visualization in the present study. However, 3D visualization helped compensate for the lack of experience, allowing trainees to delineate margins as accurately as or more accurately than experts under 2D visualization. As mentioned in previous studies related to 3D visualization, one of the advantages of 3D endoscopy is its usefulness among endoscopists without sufficient experience in endoscopy [22, 23].

Several limitations associated with the present study warrant mention. First, the correct lesion margins constructed based on the mapping images in the pathological diagnosis may not be exactly the same as the actual lesion extent on endoscopic observation. Second, participants were not blinded to whether they were performing 2D or 3D visualization. Third, the number of subjects was relatively small, and this study was carried out at a single institution. Further studies to investigate the usefulness of 3D flexible endoscopy for the endoscopic diagnosis of gastrointestinal neoplasias are warranted.

## Conclusions

In conclusion, 3D visualization improves the performance of the endoscopic diagnosis of superficial gastric neoplasias. These findings may improve the early detection of EGC and safe ESD without a positive margin for tumor cells.

## Supplementary Information

**Additional file 1: Supplementary Table 1**. The comparison of the coincidence rate of lesion extent in group A and group B. **Supplementary Table 2**. The comparison of the ease of endoscopic lesion recognition by the skill level of endoscopists.

## Data Availability

The datasets used and/or analyzed during the current study are available from the corresponding author on reasonable request.

## Abbreviations

ESD: Endoscopic submucosal dissection
EGC: Early gastric cancer
NBI: Narrow-band imaging
3D: 3-Dimensional
2D: 2-Dimensional
WLI: White-light imaging
VAS: Visual analog scaled
M-NBI: Magnifying endoscopy with narrow-band imaging
