# Supplementary material for: Three-dimensional visualization improves the endoscopic diagnosis of superficial gastric neoplasia
Source: BMC Gastroenterol. 2021 May 28;21:242. doi: 10.1186/s12876-021-01829-y (PMC8161972; doi:10.1186/s12876-021-01829-y)
Supplement: Supplementary file 1 — Additional file 1: Supplementary Table 1. The comparison of the coincidence rate of lesion extent in group A and group B. Supplementary Table 2. The comparison of the ease of endoscopic lesion recognition by the skill level of endoscopists. [file 12876_2021_1829_MOESM1_ESM.pdf]

Supplementary information

Three-dimensional visualization improves the endoscopic diagnosis of superficial gastric neoplasia

Kazutoshi Higuchi[1], Mitsuru Kaise[1], Hiroto Noda[1], Kumiko Kirita[1], Eriko Koizumi[1], Takamitsu Umeda[1], Teppei Akimoto[1], Jun Omori[1], Naohiko Akimoto[1], Osamu Goto[1], Atsushi Tatsuguchi[1, 2], Katsuhiko Iwakiri[1]

[1]Department of Gastroenterology, Nippon Medical School, Graduate School of Medicine, Tokyo, Japan

[2]Department of Analytic Human Pathology, Nippon Medical School, Graduate School of Medicine, Tokyo, Japan

Supplementary Table 1. The comparison of the coincidence rate of lesion extent in group A and group B

		group A	group B	*P* value
WLI	2D	51.4% (45.6 – 57.1)	53.2% (47.2 – 59.3)	0.299
	3D	60.7% (55.0 – 66.4)	59.7% (53.7 – 65.6)	0.965
NBI	2D	63.7% (58.9 – 68.4)	63.8% (58.8 – 68.9)	0.861
	3D	70.8% (66.0 – 75.6)	69.2% (64.0 – 74.3)	0.480

Data are presented as the mean (95% confidence interval).

WLI, white-light imaging; NBI, narrow-band imaging; 2D, 2-dimensional; 3D, 3-dimensional

Supplementary Table 2. The comparison of the ease of endoscopic lesion recognition by the skill level of endoscopists

		WLI		*P* value	NBI		*P* value
		**2D**	**3D**		**2D**	**3D**	
lesion morphology	Novice	3.23 (2.62)	5.50 (3.56)	<0.001	4.43 (2.49)	6.57 (3.05)	<0.001
	Trainee	6.03 (2.59)	8.04 (1.91)	<0.001	6.49 (1.80)	8.38 (1.79)	<0.001
	Expert	5.09 (2.27)	7.54 (2.11)	<0.001	6.63 (1.77)	8.08 (1.43)	<0.001
lesion extent	Novice	2.18 (2.00)	4.17 (2.99)	<0.001	3.17 (2.33)	5.13 (2.89)	<0.001
	Trainee	5.50 (2.65)	7.11 (2.45)	<0.001	5.77 (2.26)	7.37 (2.17)	<0.001
	Expert	4.87 (2.36)	6.88 (2.09)	<0.001	6.28 (1.63)	7.79 (1.47)	<0.001
comprehensive	Novice	2.27	4.06	<0.001	3.40	5.61	<0.001

endoscopic		(2.45)	(3.29)		(2.49)	(3.29)	
cognition	Trainee	5.71	7.40	<0.001	6.26	7.97	<0.001
		(2.43)	(2.10)		(1.81)	(1.72)	
	Expert	4.87	7.18	<0.001	6.36	7.96	<0.001
		(2.35)	(2.12)		(1.60)	(1.41)	

Data are presented as the mean (standard deviation).

WLI, white-light imaging; NBI, narrow-band imaging; 2D, 2-dimensional; 3D, 3-dimensional
